# Superwetting membrane-based strategy for high-flux enrichment of ethanol from ethanol/water mixture

**DOI:** 10.3389/fchem.2022.1037828

**Published:** 2022-09-30

**Authors:** Zhongwei Wei, Shaoqing Zhang, Li Chang, Hongliang Liu, Lei Jiang

**Affiliations:** ^1^ Key Laboratory of Bio-inspired Materials and Interfacial Science, Technical Institute of Physics and Chemistry, Chinese Academy of Sciences, Beijing, China; ^2^ School of Future Technology, University of Chinese Academy of Sciences, Beijing, China; ^3^ School of Chemistry and Chemical Engineering, Yantai University, Yantai, China; ^4^ Key Laboratory of Bio-inspired Smart Interfacial Science and Technology of Ministry of Education, School of Chemistry, Beihang University, Beijing, China

**Keywords:** superwetting, membrane separation, high flux, ethanol/water separation, bioethanol

## Abstract

Ethanol, which can be scalable produced from fermented plant materials, is a promising candidate to gasoline as the next-generation liquid fuel. As an energy-efficient alternative to distillation, membrane-based strategies including pervaporation and reverse osmosis have been developed to recover ethanol from fermentation broths. However, these approaches suffer the drawback of low separation flux. Herein, we report a superwetting membrane system to enrich ethanol from water in a high-flux manner. By synergistically regulating surface energy of the solid porous membrane and hydration between an additive inorganic potassium salt and water, concentrated ethanol can rapidly wetting and permeate the porous membrane, with the salt solution being blocked. Using this newly developed superwetting membrane system, we can achieve fast enrichment of ethanol from water, with flux of two orders magnitude higher than that of pervaporation and reverse osmosis membranes.

## 1 Introduction

More-sustainable fuels are being intensively searched for to replace non-renewable fossil fuels. In this context, biofuels, such as bioethanol, produced by fermenting plant materials could provide alternative fuels (Frolkova and Raeva, 2010; Baeyens et al., 2015; Singh and Rangaiah, 2017; Khalid et al., 2019). In US and Brazil, it is quite common that ethanol is used in gasohol with 90% gasoline, 10% ethanol (Hartline, 1979). It is estimated that demand for ethanol will grow by 5.2% annually, reaching 14.6 billion gallons by 2030 (Carolan, 2009). Generally, ethanol in the fermentation broth is in the range of 4 wt% to 12 wt% (Singh and Rangaiah, 2017). Therefore, it is necessary to gain ethanol with high purity before it can be used as liquid fuel. Distillation is the most used separation technique, but is energy-consuming (Frolkova and Raeva, 2010; Chávez-Islas et al., 2011; García-Herreros et al., 2011; Singh and Rangaiah, 2017; Khalid et al., 2019). With the rapid development of membrane separation technology, pervaporation and reverse osmosis membranes are considered to be more cost saving for ethanol purification (Mulder et al., 1983; Choudhury et al., 1985; Lee et al., 1985; Frolkova and Raeva, 2010; Peng et al., 2010; Khalid et al., 2019). For example, polystyrene-grafted cellulose acetate membranes have been used for successful separation of ethanol/water mixture by reverse osmosis (Choudhury et al., 1985). However, pervaporation and reverse osmosis membranes that relying on the solution-diffusion model are inherently limited by their low separation flux. In contrast, superwetting membranes that depending on the capillary force of micrometer pores can efficiently separate immiscible liquids mixture with far higher flux than other membrane-based separations (Feng et al., 2004; Wen et al., 2013; Zhang et al., 2013; Wang et al., 2015; Wang et al., 2017; Tang et al., 2020; Cai et al., 2021; Zhang et al., 2022). However, it has long been a challenging task to separate miscible liquids mixture by using high-flux superwetting membranes, until we recently made a breakthrough by combinational introduction of an extra inductive agent and surface energy regulation of the solid membrane (Chang et al., 2022). Inspired by this idea, could we apply this design principle for high-flux separation of ethanol and water?

Herein, we report a superwetting membrane system (SMS) with the capability to enrich ethanol from ethanol/water mixture in a high-flux manner. An inorganic potassium salt that can form strong hydration with water molecules was introduced to the system. Together with synergistically tuning the surface energy of the porous membrane, ethanol molecules can preferably permeate the porous membrane, with the remaining salt solution being blocked. In this way, diluted ethanol can be efficiently enriched with high flux.

## 2 Experimental sections

### 2.1 Materials

Ethanol (Sinopharm Chemical Reagent, 99.5%), acetic acid (Sinopharm Chemical Reagent, 99.5%), tetraethyl orthosilicate (TEOS, TCI (Shanghai) Chemical Trading, 97%), titanium (IV) butoxide (TNTB, J&K Scientific, 99%), polyvinylpyrrolidone (PVP, MW ∼1.3 × 10^6^, J&K Scientific), n-hexyltrimethoxysilane (J&K Scientific, 97%), 1H,1H,2H,2H-perfluorooctyltrimethoxysilane (J&K Scientific, 96%), 3-aminopropyltriethoxysilane (J&K Scientific, 98%), potassium phosphate trihydrate (K_3_PO_4_∙3H_2_O, Sinopharm Chemical Reagent, 99%), dipotassium hydrogen phosphate trihydrate (K_2_HPO_4_∙3H_2_O, J&K Scientific, 99%), potassium citrate monohydrate (K_3_C_6_H_5_O_7_∙H_2_O, J&K Scientific, 99%), Nile red (Rhawn, 95%) and anhydrous copper (II) sulfate (Shanghai Macklin Biochemical, 99%) were used as received. Potassium pyrophosphate (K_4_P_2_O_7_, Sigma-Aldrich, 97%) was dried at 105 °C under a reduced pressure of about 0.03 atm for 4 h prior to use. Potassium carbonate (K_2_CO_3_, Sinopharm Chemical Reagent, 99%) and potassium thiosulfate (K_2_S_2_O_3_, Sigma-Aldrich, 95%) were dried at 140 °C under a reduced pressure of about 0.03 atm for 4 h prior to use.

### 2.2 Fabrication of SiO_2_-TiO_2_ composite membranes (denoted as STMs)

#### 2.2.1 Fabrication of bare STMs

The fabrication process of bare STMs includes three steps (Wang et al., 2015; Chang et al., 2022).

1) Preparation of electrospinning solutions. First, 1 g of acetic acid, 4.6 ∼ 5.2 g of ethanol and 0.8 ∼ 1.4 g of PVP were mixed and stirred for 0.5 h. The total mass of ethanol and PVP was 6 g. Next, 2.3 g of TEOS and 0.7 g of TNTB were dripped into the mixture with stirring for 24 h at room temperature to achieve homogenous solutions.
2) Process of electrospinning. The precursor solution was loaded into a plastic syringe positioned vertically with 24-Gauge blunt stainless nozzle and injected at a flow rate of 1.3 ml h^−1^ *via* a digital syringe pump. The static electric field was generated by applying positive electrical potential on the metallic needle and negative electrical potential on an aluminum foil-covered metallic rotating roller, with a minimal distance of 11 cm, and the voltage was set at 17 kV.
3) Postprocessing electrospun membranes. The finally flexible fibrous membranes were obtained by drying the electrospun membranes at 80°C for 15 h and then calcining them at 600°C for 6 h following a heating of 5°C min^−1^.

#### 2.2.2 Fabrication of STM-C_6_, STM-C_8_F_13_ and STM-NH_2_

Bare STMs were treated with plasma for 5 min at high RF level under vacuum (PDC-002, HARRICK) to generate hydroxyl groups before surface chemical modifications. The STM-C_6_, STM-C_8_F_13_ and STM-NH_2_ were prepared by silanization of bare STM with n-hexyltrimethoxysilane, 1H,1H,2H,2H-perfluorooctyltrimethoxysilane and 3-aminopropyltriethoxysilane, respectively. Specifically, the substrates were performed in a closed chamber with concentration of the gaseous reagents about 0.2 g dm^−3^ at 130°C under a reduced pressure of about 0.1 atm for 4 h.

### 2.3 Characterization of STM-C_6_, STM-C_8_F_13_ and STM-NH_2_

The successful modification of bare STM was confirmed by measuring surface elemental composition of the membranes with an ESCALAB 250Xi X-ray photoelectron spectroscopy (XPS) from ThermoFisher Scientific.

The microstructures of the STM-C_6_, STM-C_8_F_13_ and STM-NH_2_ were obtained with an SU8010 scanning electron microscope (SEM).

### 2.4 Contact angle (CA) measurements

The CAs were measured at room temperature with an OCA20 system from DataPhysics. A 0.2∼2 μl droplet of measured liquid was deposited with a 24-Gauge blunt stainless syringe onto a chemically modified STM, which was adhered to the glass substrate and under air or another liquid environment. The average CA value was obtained by measuring the CAs at 3 or more different positions on one sample.

### 2.5 Determination of ternary phase diagrams

The phase diagram for each ethanol/water/salt system was obtained from the binodal curve and eutectic points. The boundary between biphasic and triphasic zone was a line segment, determined by the two eutectic points.

The binodal curve was obtained by cloud point titration at room temperature (Wang et al., 2010; Lu et al., 2013). The salt was weighed with an analytical balance (LE204E, from METTLER TOLEDO) with an uncertainty of ±1 × 10^−7^ kg. Water was added to form a homogeneous mixture, and ethanol was dropwise added to obtain next cloud point, which was repeated until moderate ethanol content. The masses of both liquids were calculated from their densities and volumes recorded by burettes. At high ethanol content, salt solution and water were added into ethanol. Both ethanol and salt solution were defined by the analytical balance, and water was recorded by a burette. The data was correlated by the following equation to form the binodal curve (Hu et al., 2003; Wang et al., 2010):

$$w_{C_{2}H_{5}\text{OH}}^{0.4}=a+bw_{\text{salt}}^{0.25}+cw_{\text{salt}}^{0.5}+dw_{\text{salt}}+ew_{\text{salt}}^{1.5}.$$

Ethanol was added to concentrated salt solution, forming a triphasic mixture with two liquid phases in equilibrium with a solid phase. The two eutectic points were obtained by the water content and salt content of the liquid phases. Water content was determined by Karl-Fischer titration with a V20 Volumetric KF titrator from METTLER TOLEDO. Salt content was calculated from the mass fraction of potassium, which was determined with an IRIS Intrepid II XSP inductively coupled plasma atomic emission spectroscopy from ThermoFisher Scientific.

### 2.6 Separation of ethanol/water mixture

The separation of ethanol/water mixture was done with a self-made separation device. In this device, an STM-M was sandwiched between two Teflon flanges, which were installed between two reservoirs. Ethanol/water mixture and salt were added in the upper reservoir, and separated liquid can be collected in the lower reservoir.

The separation efficiency was evaluated by the purity of ethanol in the permeate, calculated from the mass fractions of water and salt as:

$$w_{C_{2}H_{5}\text{OH}}=1-w_{H_{2}O}-w_{\text{salt}}.$$

The flux was determined by measuring the mass of the permeate within certain time, calculated with the formula:

$$q=\frac{m}{\rho At},$$

where q is the flux, m is the mass of permeate within time t , ρ is the density of permeate, and A is the effective area of the STM-M.

## 3 Results and discussion

### 3.1 Design of the SMS for enrichment of ethanol from ethanol/water mixture

**FIGURE 1:**
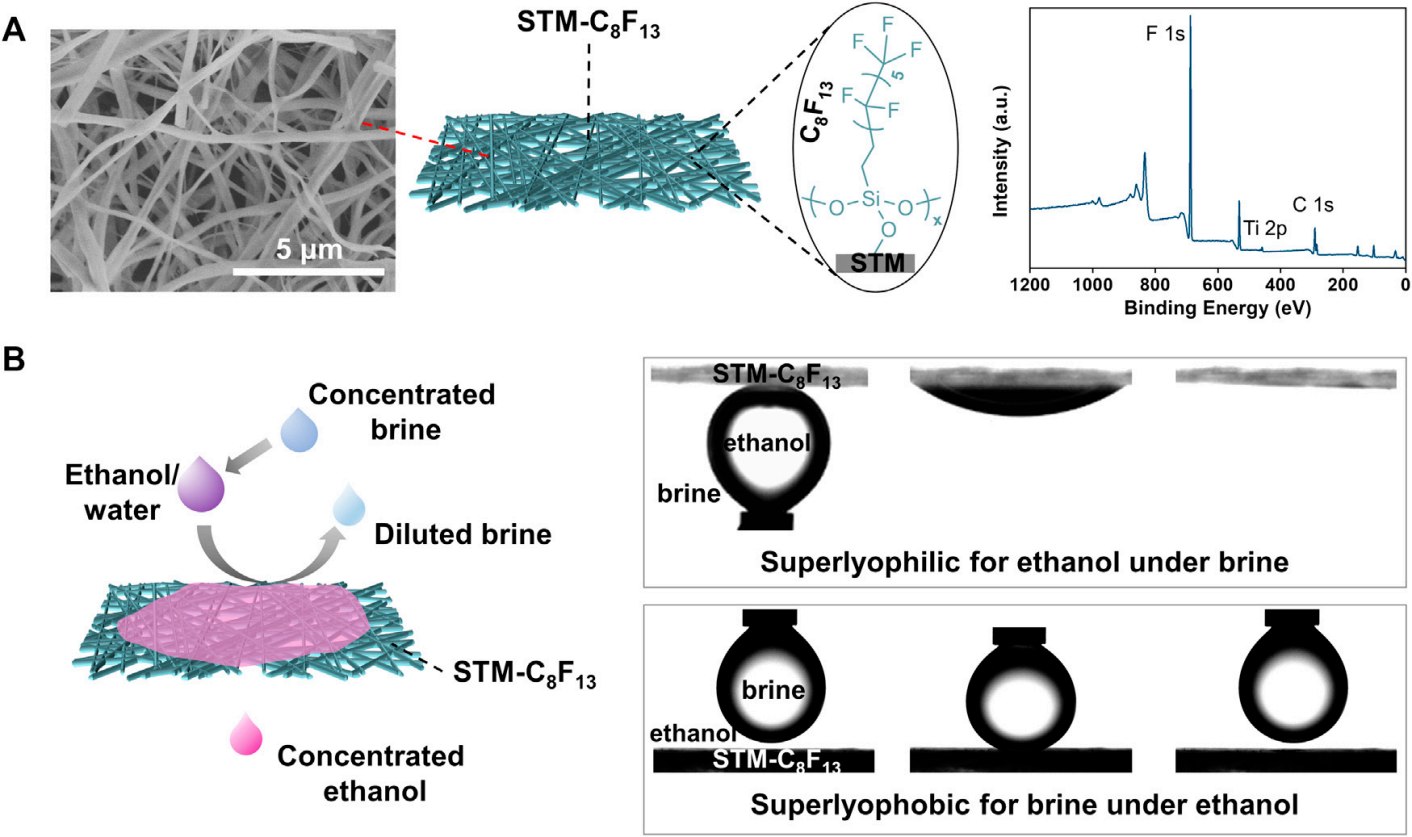
Design of SMSs for ethanol/water separation. **(A)** SEM image, chemical structure and XPS spectrum of the STM-C_8_F_13_. **(B)** Scheme and dynamic wetting behaviors of superwetting membrane-based separation in the presence of saturated K_4_P_2_O_7_ as an inductive agent.

Porous membranes with proper surface energies were prepared by chemical modification of inorganic STMs generated by electrospinning a viscous precursor solution and subsequent high-temperature calcination. The STMs are composed of entangled nanofibers forming multilayer networks with random pores (left in Figure 1A). Then -CF_3_ terminated silane coupling agent 1H,1H,2H,2H-perfluorooctyltrimethoxysilane was introduced onto the STM surface (denoted as STM-C_8_F_13_) (middle in Figure 1A). X-ray photoelectron spectroscopy (XPS) data clearly show the characteristic peak of fluorine (right in Figure 1A), indicating successful modification of the STM.

In initial wetting test, static contact angles (CAs) of the ethanol/water droplets on the STM-C_8_F_13_ are concentration-dependent. For mixed droplets with ethanol concentrations of 10 wt% and 30 wt%, the STM-C_8_F_13_ is lyophobic with CAs of about 120^o^. For mixed droplets with ethanol percentage of 50 wt% and 70 wt%, the STM- C_8_F_13_ changes to superlyophilic with CAs close to zero (Supplementary Figure S1). Nevertheless, when an inductive agent, concentrated potassium pyrophosphate (K_4_P_2_O_7_) solution was introduced to the system, strong hydration between K_4_P_2_O_7_ and water molecules, together with the high affinity between C_8_F_13_ and ethanol, led to selective permeation of ethanol to obtain concentrated ethanol (left in Figure 1B). In this case, the STM-C_8_F_13_ shows superlyophilic for ethanol under K_4_P_2_O_7_ solution and superlyophobic for K_4_P_2_O_7_ solution under ethanol (right in Figure 1B). Thus, STM-C_8_F_13_ can be selectively wetted by concentrated ethanol while concurrently blocking K_4_P_2_O_7_ solution. Specifically, when a feed ethanol/water mixture with 10 wt% of ethanol passes through the STM-C_8_F_13_ membrane in the presence of saturated K_4_P_2_O_7_ solution, the ethanol concentration in the permeate is as high as 90.1 wt%.

### 3.2 Determination of ternary phase diagram of ethanol/water/salts

In our designed SMSs, the introduction of inorganic salts as inductive agents is significantly important to generate effective phase separation. 18 inorganic salts were used to determine the ternary phase diagram of ethanol/water/salts. The binodal curve was obtained by cloud point titration. The boundary between biphasic and triphasic zone was a line segment, determined by its eutectic points, which were compositions of two liquid phases in equilibrium with a solid phase. With high water content, ethanol and salt both dissolved, forming a homogeneous mixture. With moderate water content, phase separation happened, forming one liquid phase with high ethanol content and the other liquid phase with almost no ethanol. The composition of each phase was on the binodal curve. With low water content, solid salt crystalized from the mixture, forming three phases. The composition of each phase was fixed, independent of overall composition.

**FIGURE 2:**
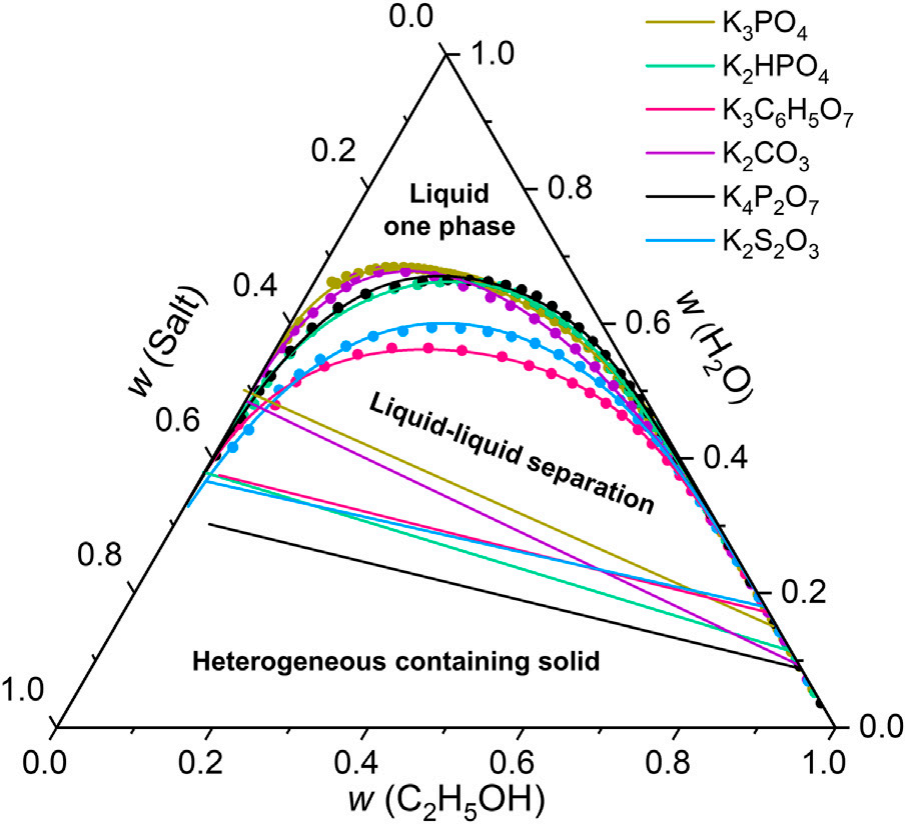
Ternary phase diagram of ethanol/water/salts. The binodal curve was obtained by cloud point titration. The boundary between biphasic and triphasic zone was a line segment, determined by its eutectic points, which were compositions of two liquid phases in equilibrium with a solid phase.

As shown in Figure 2, the binodal curves and the line segments between biphasic and triphasic zones of 6 potassium salts were obtained by using this method. However, the other 12 salts with weaker salting-out effect, including K_2_SO_4_, KHSO_4_, MgSO_4_, (NH_4_)_2_SO_4_, Na_2_SO_4_, KH_2_PO_4_, KH_2_C_6_H_5_O_7_, KHCO_3_, KCl, KBr, KI, KSCN, do not have liquid-liquid equilibrium zone, and thus not suitable for ethanol/water separation.

### 3.3 Separation performance for ethanol/water

**FIGURE 3:**
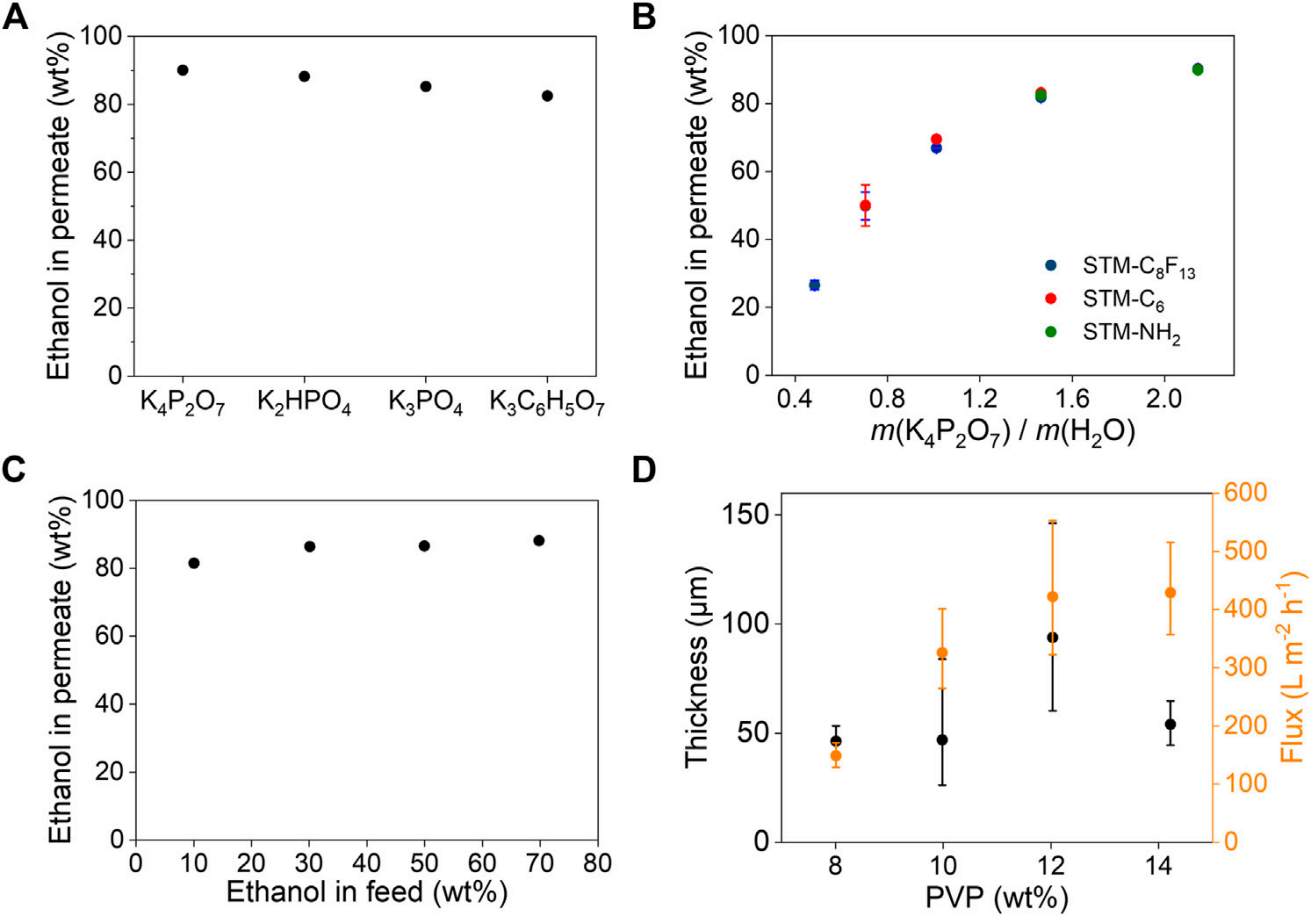
Separation performance for ethanol/water. **(A)** Separation efficiencies of SMS-C_8_F_13_ with different saturated potassium salts solutions as the inductive agents. Ethanol in feed is 10 wt%. **(B)** Separation efficiencies of three different SMSs with different amount of K_4_P_2_O_7_. **(C)** Separation efficiencies of SMS-C_8_F_13_ for ethanol/water with different compositions in the presence of saturated K_4_P_2_O_7_ solution. **(D)** Separation fluxes and thicknesses of STM-C_8_F_13_ prepared using different PVP concentrations. Ethanol in feed is 50 wt%. Inductive agent is saturated K_4_P_2_O_7_ solution. The flow was driven by the gravity of 1 cm high liquid. Error bars represent the standard deviation form at least three independent experiments.

The separation performance of SMSs for ethanol/water was studied by tuning the types of the inductive agents, chemical compositions and pore sizes of the porous membranes. The separation efficiencies depend strongly on the types of inductive agents (Figure 3A). With addition of saturated salt solutions, K_4_P_2_O_7_, K_2_HPO_4_, K_3_C_6_H_5_O_7_ and K_3_PO_4_ all resulted higher than 80 wt% concentration of ethanol in permeate. K_4_P_2_O_7_ resulted the highest concentration of 90.1% due to the highest thermodynamic limit, consistent with the results showed in phase diagrams (Figure 2). Therefore, we chose K_4_P_2_O_7_ as the optimal inductive agent for further study.

The amount of K_4_P_2_O_7_ also plays an important role in separation efficiency. As shown in Figure 3B, ethanol in permeate increases obviously with increasing concentration of K_4_P_2_O_7_, independent on the surface chemistry of the porous membranes (Supplementary Figure S2). However, porous membranes with different surface chemistry differ in thresholds of K_4_P_2_O_7_ addition (Supplementary Figure S3). The ethanol/water mixture can be separated by the membrane if more K_4_P_2_O_7_ was added than the threshold, otherwise it cannot. For example, there was a threshold point on ethanol/water/K_4_P_2_O_7_ binodal curve for the STM-C_6_. To left of the point, the liquid was blocked by STM-C_6_; to right of the point, the liquid would permeate STM-C_6_. So, if the composition of biphasic mixture was above the red line, both phases were blocked by STM-C_6_, not separated. Only if the composition of biphasic mixture was below the red line, STM-C_6_ can separate it. Similarly, for the STM-NH_2_, if the composition of biphasic mixture was above the green line, both phases would permeate STM-NH_2_, not separated. In contrast, for STM-C_8_F_13_, there is no such threshold and ethanol can be separated from the mixture as long as phase separation occurs. So, STM-C_8_F_13_ was selected for enrichment of ethanol from a wide range of ethanol/water feed compositions. Ethanol concentration in feed slightly affected ethanol concentration in permeate, but all achieved a higher concentration than 80 wt% (Figure 3C).

The separation flux of SMSs was further investigated by regulating the microstructures of the porous membranes (Supplementary Figure S4). By controlling the PVP concentrations during preparation of the porous membranes by electrospinning. As shown in Supplementary Figure S4, with increasing concentration of PVP, diameters of the nanofibers become larger and networks of the porous membranes get looser, which would facilitate permeation of concentrated ethanol and thus lead to higher flux. When PVP concentration is 12 wt%, separation flux for concentrated ethanol is up to 400 L m^−2^ h^−1^ (Figure 3D), nearly 2-3 orders of magnitude higher than that of reverse osmosis and pervaporation.

### 3.4 Demonstration of the ethanol/water separation process

**FIGURE 4:**
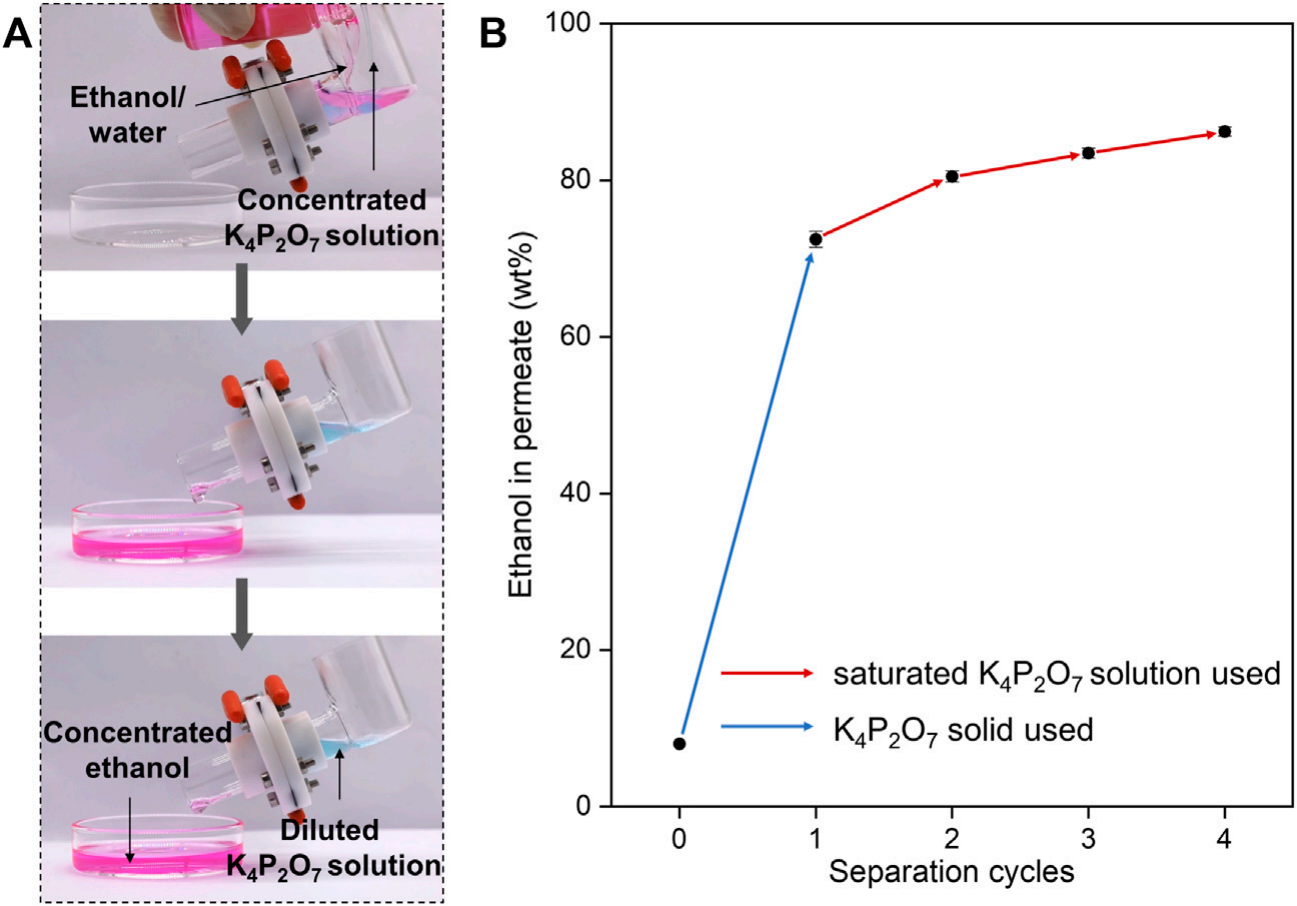
Demonstration of the ethanol/water separation. **(A)** Video captures of separation process using concentrated K_4_P_2_O_7_ solution as the inductive agent. Introducing concentrated K_4_P_2_O_7_ solution to the ethanol/water results in selective permeation and collection of ethanol with K_4_P_2_O_7_ solution being blocked. **(B)** Cycled separation for ethanol/water. Started ethanol in feed is 8 wt% and equal mass of solid K_4_P_2_O_7_ was added for separation. Each cycle takes the permeate of previous cycle as the new feed, and equal mass of saturated K_4_P_2_O_7_ solution was added.

We demonstrate the enrichment of ethanol from ethanol/water mixtures by a self-made device (Figure 4A). In this device, the STM-C_8_F_13_ was sandwiched between two Teflon flanges, which were fixed between two reservoirs. Inductive agent (saturated K_4_P_2_O_7_ solution) and ethanol/water mixture (with 10 wt% of ethanol) were added in one reservoir, and concentrated ethanol can be collected in another reservoir. To better monitor the separation process, the ethanol/water mixture and inductive agent were dyed with Nile red and anhydrous copper (II) sulfate. Pink concentrated ethanol accumulated from the mixture and permeate the STM-C_8_F_13_ in time and be collected with blue diluted K_4_P_2_O_7_ solution being blocked.

As mentioned above, ethanol concentration in permeate becomes lower with decreasing ethanol concentration in feed. This tendency becomes obvious especially when ethanol concentration in feed is lower than 10 wt%. For example, for ethanol/water mixture with 8 wt% ethanol, under optimal conditions, ethanol concentration in permeate is only 72.4 wt%. Fortunately, we can solve this problem by conducting multiple SMS-based separation processes in series. After 4 separation cycles, 86.2 wt% of ethanol in permeate was obtained (Figure 4B). Moreover, our designed SMS is compatible with traditional reverse osmosis and pervaporation membranes. Composite membrane modules composed of SMS and pervaporation are ongoing in our lab, and ethanol with higher purity could be obtained.

## 4 Conclusion

In summary, we have designed a superwetting membrane-based strategy for high-flux enrichment of ethanol from ethanol/water mixtures by synergistically tuning the inductive agents and surface energy of the porous membranes. Using this strategy, concentrated ethanol higher than 80 wt%, and separation flux as high as 400 L m^−2^ h^−1^ have been achieved. Moreover, our designed SMS can be easily assembled with conventional membranes, such as pervaporation membranes, hold the promise for the application of ethanol separation with high purification. It is believed that our work will provide an opportunity for highly efficient separation of ethanol, and would make a step forward for extended application of liquid biofuels.

## Data Availability

The raw data supporting the conclusions of this article will be made available by the authors, without undue reservation.
