# Supplementary material for: Superwetting membrane-based strategy for high-flux enrichment of ethanol from ethanol/water mixture
Source: Front Chem. 2022 Sep 30;10:1037828. doi: 10.3389/fchem.2022.1037828 (PMC9561090; doi:10.3389/fchem.2022.1037828)
Supplement: Supplementary file 1 [file DataSheet1.docx]

Superwetting Membrane-Based Strategy for High-Flux Enrichment of Ethanol from Ethanol/Water Mixture

Zhongwei Wei^1,3^, Li Chang^4^, Hongliang Liu^2*^, Lei Jiang^1,2,3,4*^

^1^Key Laboratory of Bio-inspired Materials and Interfacial Science, Technical Institute of Physics and Chemistry, Chinese Academy of Sciences, Beijing 100190, P. R. China

^2^School of Chemistry and Chemical Engineering, Yantai University, Yantai 264005, P. R. China

^3^School of Future Technology, University of Chinese Academy of Sciences, Beijing 100049, P. R. China

^4^Key Laboratory of Bio-inspired Smart Interfacial Science and Technology of Ministry of Education, School of Chemistry, Beihang University, Beijing 100191, P. R. China


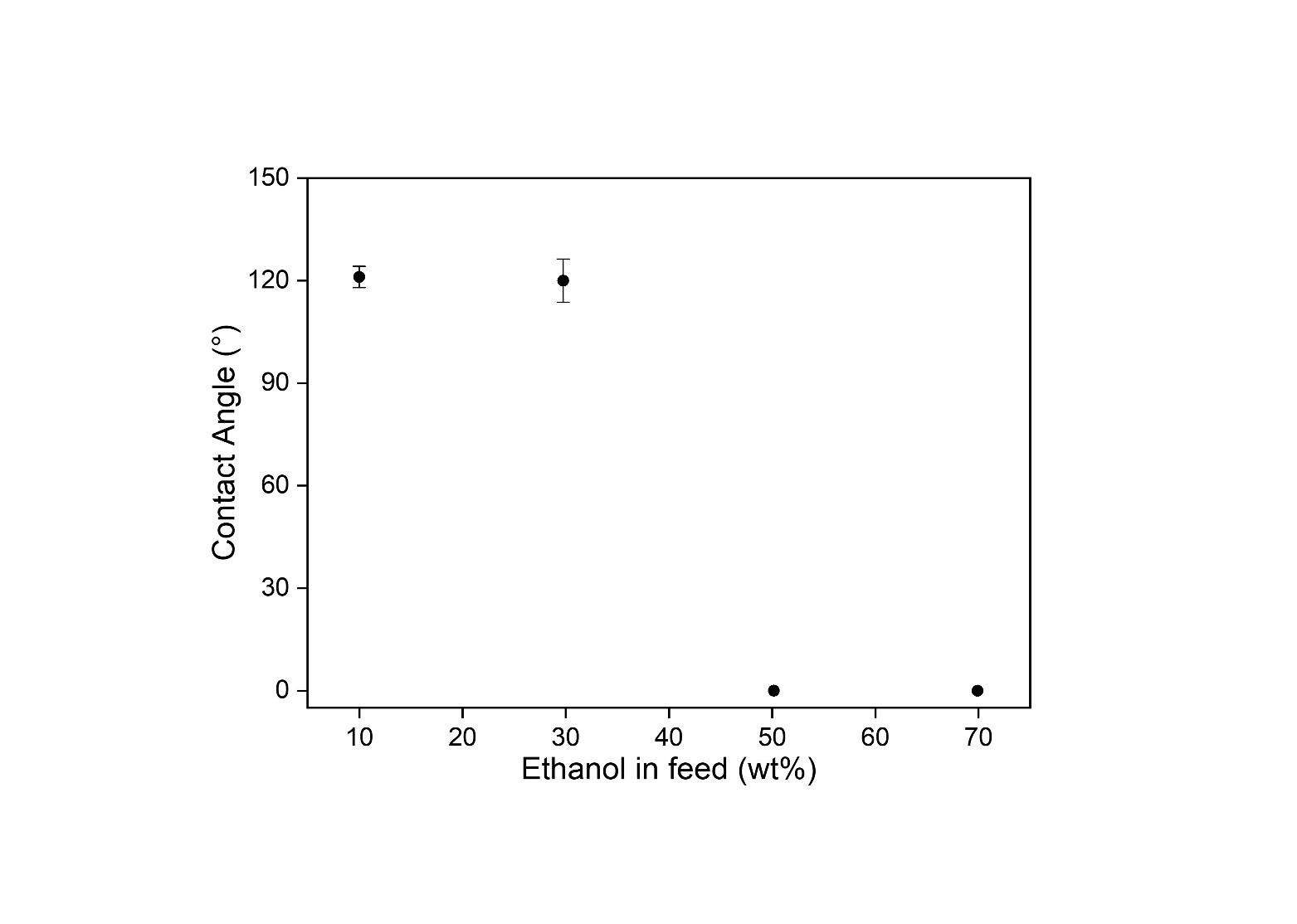


FIGURE S1. Contact angles of ethanol/water droplets with different compositions on STM-C_8_F_13_ in the absence of inductive agents.


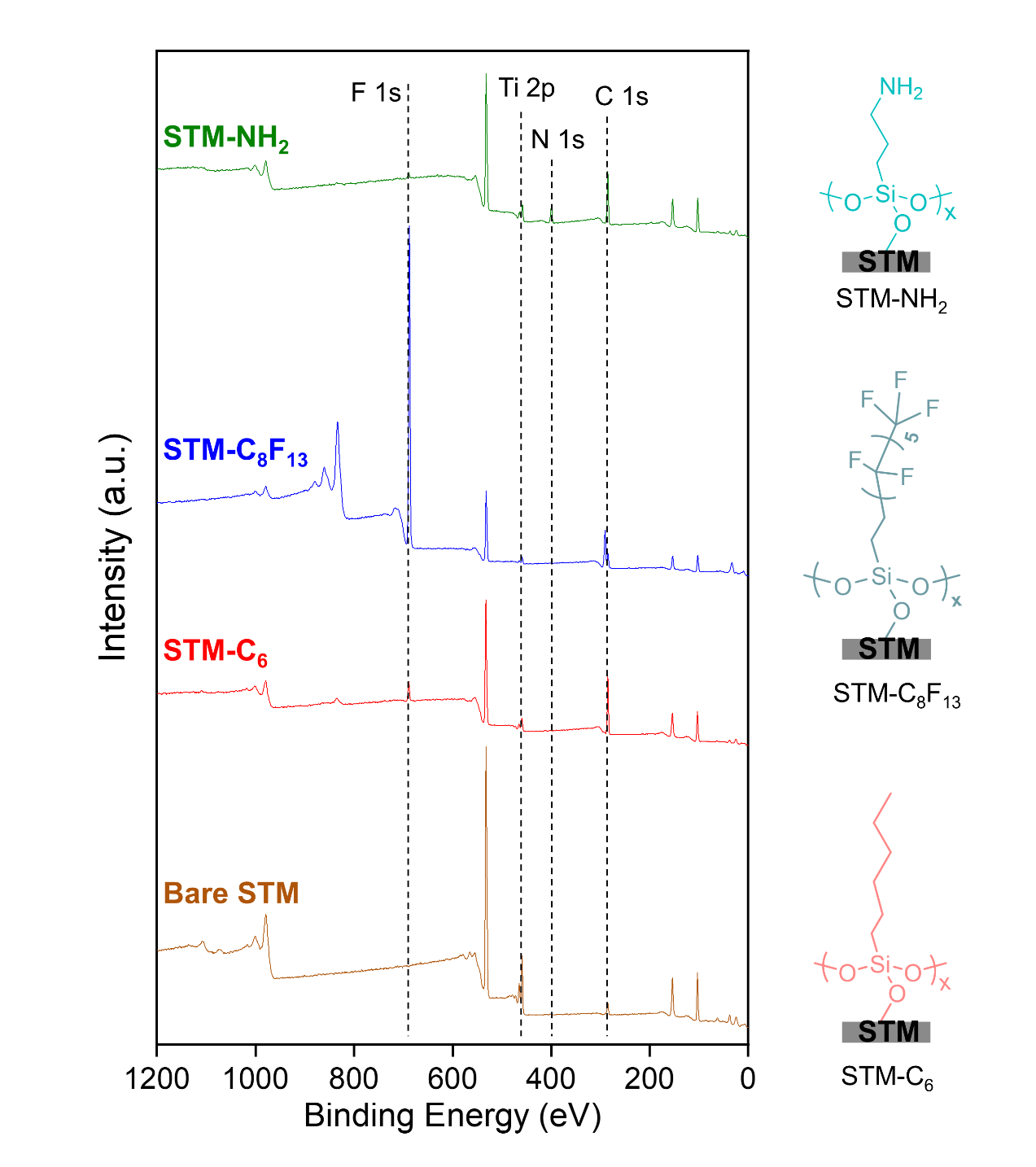


FIGURE S2. XPS spectra of bare STM and other three membranes with different surface chemistries.


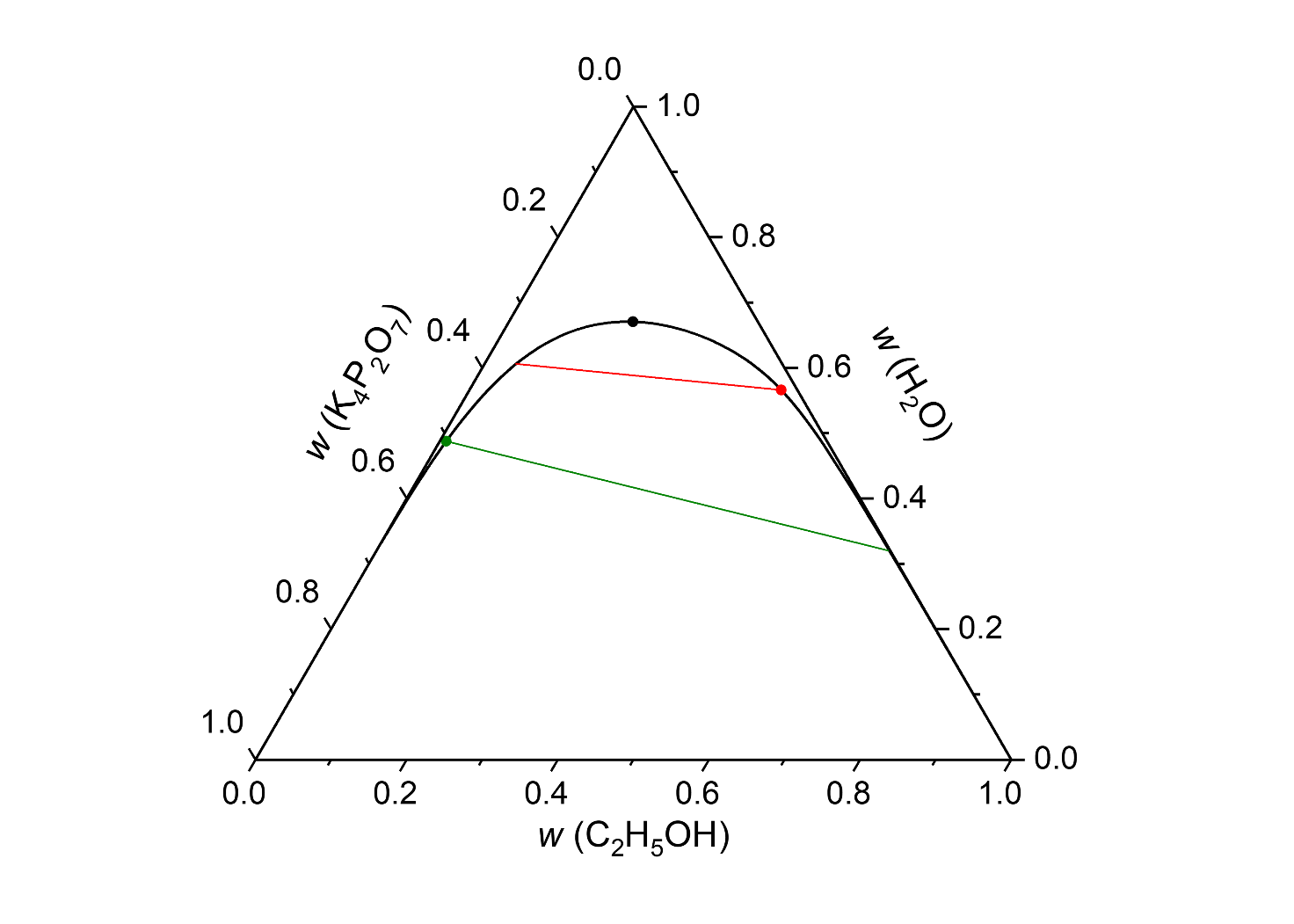


FIGURE S3. Thresholds for STM-C_6_ (red) and STM-NH_2_ (green). For STM-C_6_, only if the composition of biphasic mixture was below the red line, successful separation can occur. Otherwise, both phases were blocked by STM-C_6_. For STM-NH_2_, only if the composition of biphasic mixture was below the green line, successful separation can occur. Otherwise, both phases would permeate STM-NH_2_.


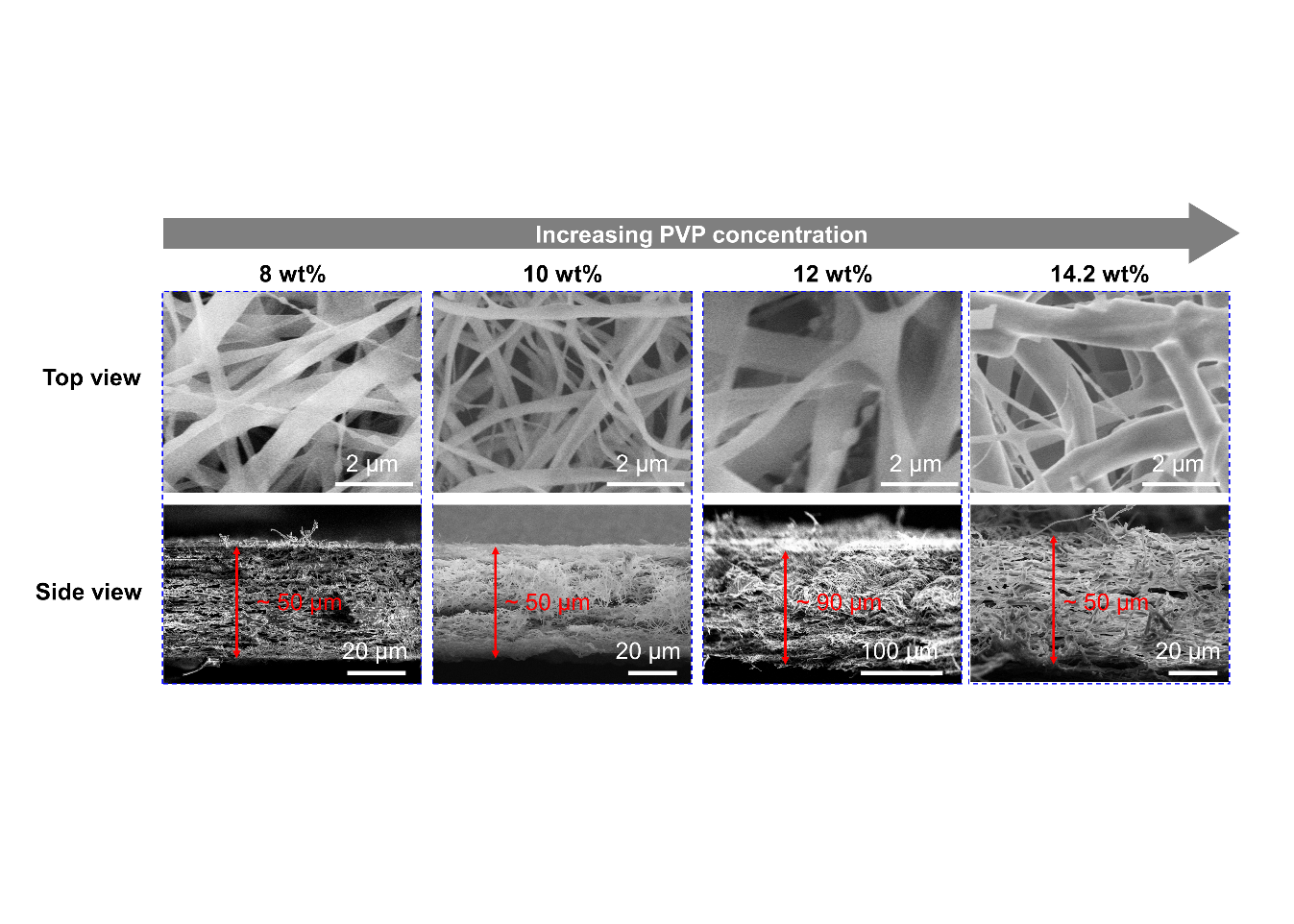


FIGURE S4. SEM images of STM-C_8_F_13_ prepared using different PVP concentrations during preparation of the porous membranes by electrospinning.
